# A Novel Immune-Related Gene Prognostic Index (IRGPI) in Pancreatic Adenocarcinoma (PAAD) and Its Implications in the Tumor Microenvironment

**DOI:** 10.3390/cancers14225652

**Published:** 2022-11-17

**Authors:** Shujing Zhou, Attila Gábor Szöllősi, Xufeng Huang, Yi-Che Chang-Chien, András Hajdu

**Affiliations:** 1 Department Data Science and Visualization, Faculty of Informatics, University of Debrecen, 4028 Debrecen, Hungary; 2 Faculty of Medicine, University of Debrecen, 4032 Debrecen, Hungary; 3 Department of Immunology, Faculty of Medicine, University of Debrecen, 4032 Debrecen, Hungary; 4 Faculty of Dentistry, University of Debrecen, 4032 Debrecen, Hungary; 5 Department of Pathology, Faculty of Medicine, University of Debrecen, 4032 Debrecen, Hungary

**Keywords:** pancreatic cancer, machine learning, gene signature, molecular subtypes, tumor microenvironment

## Abstract

**Simple Summary:**

Pancreatic adenocarcinoma (PAAD) is one of the leading causes of cancer death across the world, with extremely poor clinical outcomes within 5 years. From that end, survival prediction for such patients is essential, while in-service biomarkers are in need to be improved. Therefore, in the present study, we developed a machine learning-based prognostic model for robust and accurate survival prediction for PAAD patients. Additionally, we explored its critical implications in the tumor immunological microenvironment, sharing new insights into new therapeutic strategies in the future.

**Abstract:**

Purpose: Pancreatic adenocarcinoma (PAAD) is one of the most lethal malignancies, with less than 10% of patients surviving more than 5 years. Existing biomarkers for reliable survival rate prediction need to be enhanced. As a result, the objective of this study was to create a novel immune-related gene prognostic index (IRGPI) for estimating overall survival (OS) and to analyze the molecular subtypes based on this index. Materials and procedures: RNA sequencing and clinical data were retrieved from publicly available sources and analyzed using several R software packages. A unique IRGPI and optimum risk model were developed using a machine learning algorithm. The prediction capability of our model was then compared to that of previously proposed models. A correlation study was also conducted between the immunological tumor microenvironment, risk groups, and IRGPI genes. Furthermore, we classified PAAD into different molecular subtypes based on the expression of IRGPI genes and investigated their features in tumor immunology using the K-means clustering technique. Results: A 12-gene IRGPI (FYN, MET, LRSAM1, PSPN, ERAP2, S100A1, IL20RB, MAP3K14, SEMA6C, PRKCG, CXCL11, and GH1) was established, and verified along with a risk model. OS prediction by our model outperformed previous gene signatures. According to the findings of our correlation studies, different risk groups and IRGPI genes were found to be tightly related to tumor microenvironments, and PAAD could be further subdivided into immunologically distinct molecular subtypes based on the expression of IRGPI genes. Conclusion: The current study constructed and verified a unique IRGPI. Furthermore, our findings revealed a connection between the IRGPI and the immunological microenvironment of tumors. PAAD was differentiated into several molecular subtypes that might react differently to immunotherapy. These findings could provide new insights for precision and translational medicine for more innovative immunotherapy strategies.

## 1. Introduction

Despite the rapid development of modern therapeutic interventions in cancer progression, improvement in pancreatic adenocarcinoma (PAAD) survival is still limited, with less than 10% of patients surviving 5 years after the discovery of the tumor [1,2,3]. Currently, surgery and chemotherapy remain the mainstream in PAAD treatment [4,5,6,7,8]. Since such interventions result in high morbidity and mortality, enrichment of the arsenal of translational medicine is urgently needed [7,9].

The situation, however, is not encouraging. Although in clinical observations and animal modeling a handful of validated biomarkers such as KRAS, TP53, SMAD4, and CDKN2A, were found to exert positive effects on tumorigenesis, metastasis, and concomitantly poor prognosis, they were still believed to be insufficient to cause the disease and cannot be effectively utilized in targeting drug discovery [10,11,12,13,14,15,16].

Due to the development of bioinformatic analytics, gene signatures identified from diverse representative gene sets of well-defined tumor stages are increasingly accepted as novel tumor marker candidates. In the most recent decade, various newly defined cell death mechanisms such as ferroptosis and pyroptosis are expected to offer new insights into cancer development and cures [17,18,19]. Therefore, gene signatures derived from these two groups for prognostic prediction are “hot” research topics. On the other hand, tumor immunology, serving as the basis for immunotherapy which is proven to be successful in certain cancer types, was investigated to a much more limited degree in gene signature development, particularly in PAAD [20,21]. Therefore, the present study established a novel immune-related gene prognostic index (IRGPI) for overall survival (OS) estimation for PAAD patients, and explored more precise molecular subtypes in PAAD, together with corresponding immune characteristics.

**Figure 1:**
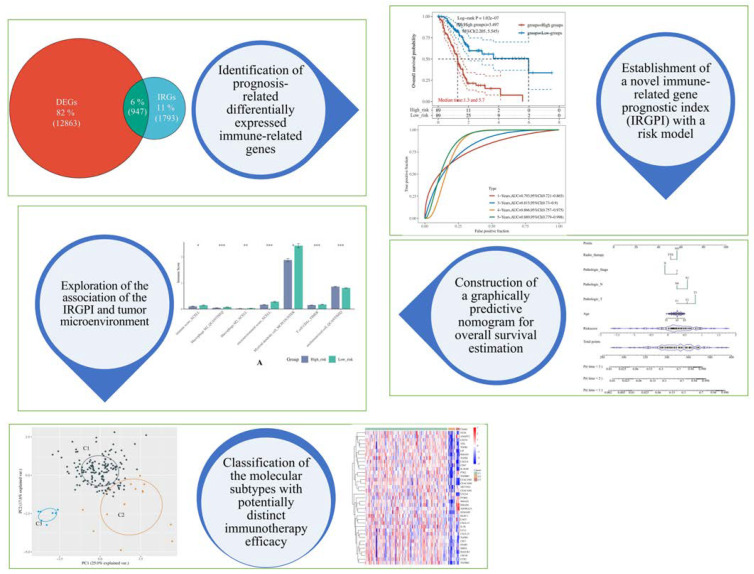
The graphical abstract of the present study. In this work, we first screened the prognosis-related differentially expressed immune-related genes in pancreatic adenocarcinoma (PAAD) to select feature genes (i.e., the 12 genes within the immune-related gene prognostic index) to construct a machine learning model. Then, we compared its predictive performance with previous models derived from ferroptosis and pyroptosis, finding that our model performed better. Next, we integrated other clinical factors such as age, pathological stage, as well as others to build a nomogram so that clinicians could use it graphically. Afterward, we explored the potential relationship between our model and the tumor microenvironment. Finally, we used unsupervised clustering to classify PAAD patients into 3 more precise molecular subtypes according to the expression of the IRGPI genes. * *p* < 0.01, ** *p* < 0.001, *** *p* < 0.0001.

The following demonstrates the workflow of the present study (Figure 1).

## 2. Materials and Methods

### 2.1. Data Acquisition and Processing

The RNA-seq data and corresponding clinical information of TNBC patients were downloaded from The Cancer Genome Atlas (TCGA) cohort (https://www.genome.gov/Funded-Programs-Projects/Cancer-Genome-Atlas (accessed on 18 March 2022)), International Cancer Genome Consortium (ICGC) data portal (https://dcc.icgc.org/ (accessed on 18 March 2022)), and Gene Expression Omnibus (GEO) database (https://www.ncbi.nlm.nih.gov/geo/ (accessed on 18 March 2022)). The well-organized data was divided into a TCGA training set (*n* = 177), an ICGC validation set (from Australian patients, *n* = 269), and a GEO validation set (from the GSE62452 dataset, *n* = 130) [22,23]. Additionally, 54 non-tumorous samples from the Genotype-Tissue Expression (GTEx) project (https://gtexportal.org (accessed on 18 March 2022)) and unique immune-related genes (IRGs) from the ImmPort database (https://www.immport.org (accessed on 18 March 2022)) were also integrated into the analytics [24,25]. The R software packages involved in the present study were embedded in R Foundation (v 4.0.3) and the GSCA platform (http://bioinfo.life.hust.edu.cn/GSCA/ (accessed on 18 March 2022)) [26]. Notably, if it wasn’t specifically mentioned, P-value < 0.05 is considered statistically significant and might be annotated as * within the figures. Moreover, **, ***, and **** might appear within the figures to indicate the *p*-value thresholds 0.01, 0.001, and 0.0001, respectively.

### 2.2. Identification of Prognosis-Related Differentially Expressed Immune-Related Genes (DEIRGs)

The R package “limma” was used to identify differentially expressed genes (DEGs) [27]. The volcano plot was plotted by R package “ggplot2” to visualize the DEGs [28]. Furthermore, the Venn diagram was drawn by JVenn to demonstrate the number and percentage of differentially expressed immune-related genes (DEIRGs) [29]. Log-rank tests and univariate Cox proportional hazard regression were conducted to screen the prognosis-related DEIRGs.

### 2.3. Establishment of Immune-Related Gene Prognostic Index (IRGPI) and Risk Model

The Least Absolute Shrinkage and Selection Operator (LASSO) penalized Cox proportional hazards regression was applied to train the optimal risk model by the R package “glmnet” [30]. The calculation of the risk score was completed after the removal of overfitting. The risk score was calculated by the following formula:

(1) $$Risk\;score=\overset{n}{\underset{i=1}{\sum }}\left(Exp_{i}\times coef_{i}\right),$$

where *n* is the number of prognostic genes, *Exp_n_* is the expression level of a specific gene, and *coef_n_* is the regression coefficient of a specific gene in the multi-Cox regression. The patients were further divided into high- and low-risk groups according to the median value of the risk score. The Kaplan Meier (KM) survival curves for OS analysis were also plotted as well as the time-dependent receiver operating characteristic (ROC) curve (for 1-, 3-, and 5-year survival) through R packages “survival” and “timeROC” [31,32].

### 2.4. Comparison with Previously Proposed Predictors

We compared the predictive performance of previous risk models derived from ferroptosis and pyroptosis in the TCGA training and ICGC validation sets. Of note, due to the lack of statistical significance between the high- and low-risk groups classified by both models in the GSE62452 dataset, such analytics could not be conducted. As a complement, we also employed the decision curve analysis (DCA) that was carried out by R package “rmda” to compare the clinical value of each model.

### 2.5. Construction of Predictive Nomogram According to the Risk Model

Combined with multiple clinical factors (i.e., age, genders, TNM stages, pathological stages, histological grades, radiotherapy), a univariate and multivariate Cox regression was performed. Moreover, according to the risk model, we further constructed a nomogram that contains only prognosis-related factors, through which the OS rate in given years could be estimated graphically.

### 2.6. Assessment of the Immunological Tumor Microenvironment

The general appearance of the tumor microenvironment was assessed by the R package “ESTIMATE” [33], while for the analysis of the abundance of various immune cell types, we curated relevant data from Tumor IMmune Estimation Resource 2.0 (TIMER 2.0) (http://timer.cistrome.org/ (accessed on 18 March 2022)) portal and processed it by the R package “immunedeconv” through 6 algorithms (i.e., TIMER, XCELL, MCPCOUNTER, CIBERSORT, EPIC, and QUANTISED) [33,34,35,36].

### 2.7. Unsupervised Consensus Clustering

According to the IRGPI, the samples of the TCGA training set were divided into subgroups utilizing consensus clustering with the R package “ConsensusClusterPlus” [37]. Then, we checked the expression of IRGPI genes in each molecular subtype to further confirm their eligibility as classifying standards. Each subtype was followed by survival analysis.

## 3. Results

### 3.1. 12 Feature Genes Were Selected to Construct the Prognosis Predictor through the LASSO Algorithm

**Figure 2:**
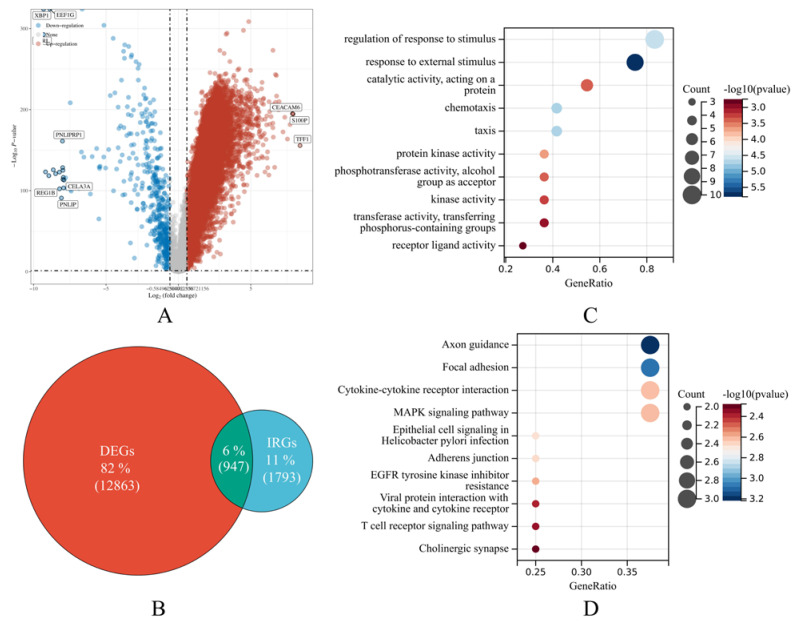
Feature gene selection and overrepresentation analysis outcomes. (**A**) The volcano plot displays the differentially expressed genes (DEGs) in pancreatic adenocarcinoma (PAAD) according to the TCGA data. The spots represent genes. The top 10 DEGs were labeled. Red shows up-regulated expression in tumorous tissues than normal samples, whereas blue represents the reverse. Non-DEGs are in gray. (**B**) The Venn diagram demonstrates the intersection of interest. The green region of the Venn diagram shows the number and ratio of DEIRGs. The red and blue sections show DEGs and immune-related genes (IRGs), respectively. (**C**,**D**) The bubble plots show gene ontology (GO) and Kyoto encyclopedia of genes and genomes (KEGG) pathway over-representation analyses. The size of the bubble corresponds to the number of genes from our samples found in the GO or KEGG pathway. *p*-value < 0.05 and FDR < 0.05 are statistically significant.

In total, 177 PAAD samples from the TCGA cohort and 54 normal samples from the GTEx project were acquired for analysis giving rise to 12,863 DEGs (Figure 2A). A total of 947 DEIRGs were extracted via intersecting with the immune-related gene list provided by the ImmPort database (Figure 2B). Eventually, survival analysis revealed that 149 genes were significantly related to the OS of PAAD patients (Supplementary Figure S1).

Then, we employed the LASSO algorithm to select the feature genes to construct the prognosis predictor with the L1 norm penalized, through which 12 genes (i.e., FYN, MET, LRSAM1, PSPN, ERAP2, S100A1, IL20RB, MAP3K14, SEMA6C, PRKCG, CXCL11, and GH1) were filtered as at this point the corresponding lambda value was minimized to 0.0986 (Supplementary Figure S2).

Afterward, an over-representation analysis was conducted. The results indicated that the most enriched gene ontology (GO) term was “regulation of response to stimulus”, followed by “response to external stimulus” (Figure 2C), and the most enriched Kyoto encyclopedia of genes and genomes (KEGG) pathway was “Axon guidance”, followed by “Focal adhesion”, “Cytokine-cytokine receptor interaction”, and “MAPK signaling pathway” (Figure 2D).

In addition, the expression of each IRGPI gene in both normal and tumorous samples was compared. As a result, except for GH1, they were found relatively higher expressed in tumorous samples (Supplementary Figure S3).

### 3.2. IRGPI-Based Risk Model Demonstrated a Strong Predictive Power

After minimizing the overfitting and reaching the minimal lambda value, a mathematical formula for risk score calculation was set as follows:

Risk score = (−0.0334) × FYN + (0.2101) × MET + (−0.0956) × LRSAM1 + (−0.2048) × PSPN + (0.0343) × ERAP2 + (-0.0159) × S100A1 + (0.0664) × IL20RB + (−0.0907) × MAP3K14 + (−0.0437) × SEMA6C + (−0.0198) × PRKCG + (0.0716) × CXCL11 + (−0.3634) × GH1

**Figure 3:**
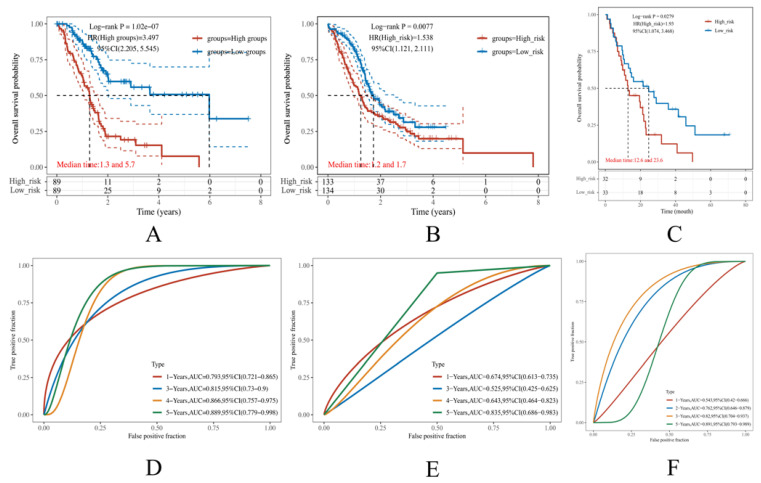
Construction of the immune-related gene prognostic index (IRGPI)-based risk model. (**A**–**C**) The Kaplan Meier (KM) curves demonstrate the overall survival (OS) rate of PAAD patients in the TCGA, ICGC, and GEO datasets, respectively. (**D**–**F**) The time-dependent receiver operative characteristics (ROC) curves of our risk model performed in the TCGA, ICGC, and GEO datasets, respectively. The larger the area under the curve (AUC), the more reliable the model.

The above formula was then applied to the ICGC and GEO validation sets. We divided the samples into high- and low-risk groups using the risk score’s median value. It turned out that the OS probability was significantly worse in high-risk patients than that in low-risk patients in all datasets. The median survival time for the high-risk group in the TCGA training set is 1.3 years, while that of the low-risk group is 5.7 years (Figure 3A). The median survival time for the high-risk group in the ICGC validation set is 1.2 years, while that of the low-risk group is 1.7 years (Figure 3B). The median survival time for the high-risk group in the GEO validation set is 12.6 months, while that of the low-risk group is 23.6 months (Figure 3C).

The AUC values in TCGA training set were 0.793, 0.815, 0.866, 0.889 in 1-, 3-, 4-, and 5-year OS (Figure 3D), and those in the ICGC validation set were 0.674, 0.525, 0.643, and 0.835 (Figure 3E). The AUC values in the GEO validation set were 0.543, 0.762, 0.82, and 0.891 in 1-, 2, 3-, and 5-year OS (Figure 3F). In general, an AUC value over 0.7 is considered a good model, and given the fact that the different database has different criteria for patient selection, the potential deviation during the validation was reasonable [38,39]. Comprehensively speaking, the result should be deemed acceptable.

### 3.3. The Predictive Performance of the IRGPI-Based Risk Model Is Superior to That of the Ferroptosis- and Pyroptosis-Derived Model

Gene signatures derived from ferroptosis and pyroptosis have recently been hot research topics. However, whether the corresponding risk models are better than ours remains unknown. For this purpose, previously proposed ferroptosis- and pyroptosis-derived risk models were tested in the TCGA, ICGC, and GEO datasets to compare with our model.

**Figure 4:**
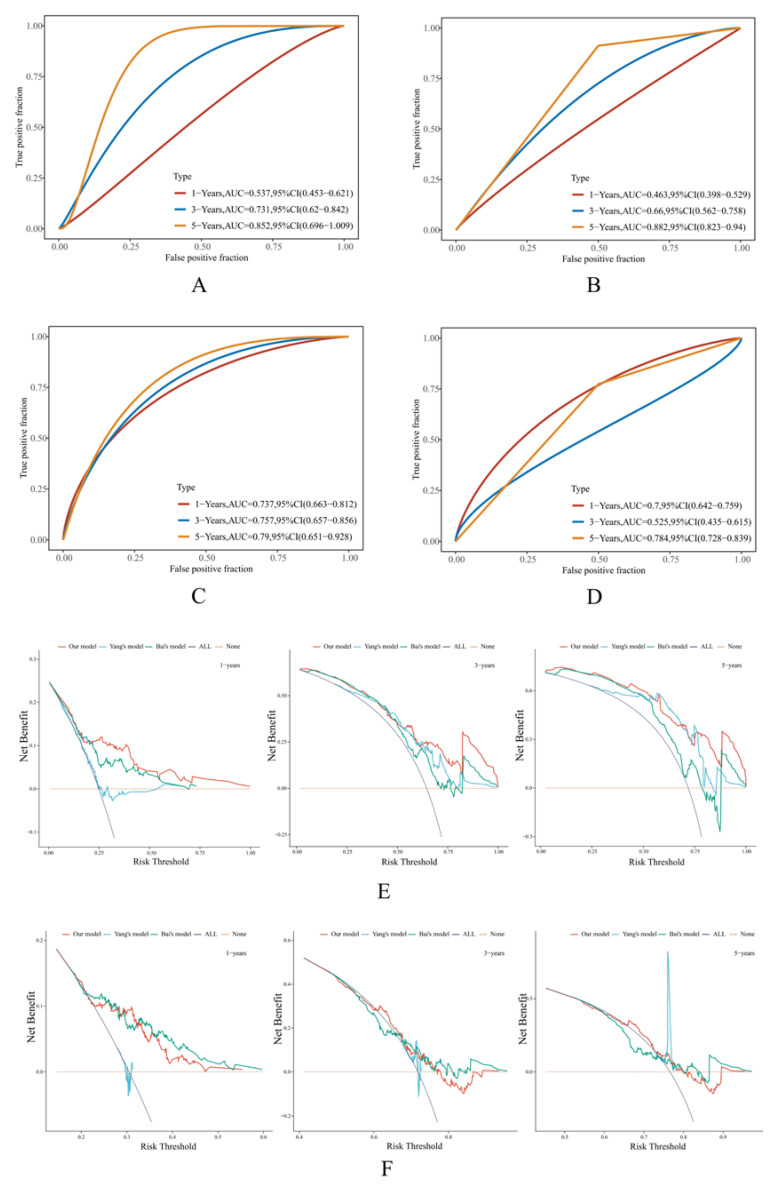
Comparison of the predictive performance of different models. (**A**,**B**) The time-dependent receiver operative characteristics (ROC) curve of Yang’s ferroptosis-derived model in the TCGA and ICGC datasets, respectively. (**C**,**D**) The time-dependent ROC curve of Bai’s pyroptosis-derived model in the TCGA and ICGC datasets, respectively. The larger the area under the curve (AUC), the more reliable the model. (**E**,**F**) The decision curve analysis (DCA) diagrams demonstrate the comparison of our risk model, Yang’s ferroptosis-derived model, and Bai’s pyroptosis-derived model in the TCGA and ICGC datasets, respectively. All: all positive; None: all negative. These serve as extreme conditions to determine background references. The distance from these extremes indicates a better clinical benefit that the corresponding model can achieve.

As a result, in the TCGA training set, the AUC values of Yang’s ferroptosis model were 0.537, 0.731, and 0.852 for 1-, 3-, and 5-year OS (Figure 4A). The AUC values of Bai’s pyroptosis model were 0.737, 0.757, and 0.79 for 1-, 3-, and 5-year OS (Figure 4C). In the ICGC dataset, the AUC values of Yang’s ferroptosis model were 0.463, 0.66, and 0.882 for 1-, 3-, and 5-year OS (Figure 4B), and those of Bai’s pyroptosis model were 0.7, 0.525, and 0.784 for 1-, 3-, and 5-year OS (Figure 4D). Notably, due to the lack of statistical significance between the high- and low-risk groups classified by both models in the GSE62452 dataset, such analytics could not be conducted, while our model still worked robustly (Figure 3F).

We additionally evaluated the predictive performance through the DCA diagram. Through the diagrams, it was observed that in the TCGA training set, the curve of our model was located superior to the others, suggesting that the corresponding performance was better (Figure 4E). In the ICGC dataset, our model functioned slightly better than the other models in 3- and 5-year OS predictions but was less convincing than Bai’s model in the 1-year OS prediction (Figure 4F).

Overall, the predictive performance of our model was considered superior to that of Yang’s and Bai’s models.

### 3.4. The Risk Score Can Serve as an Independent Prognostic Indicator

**Figure 5:**
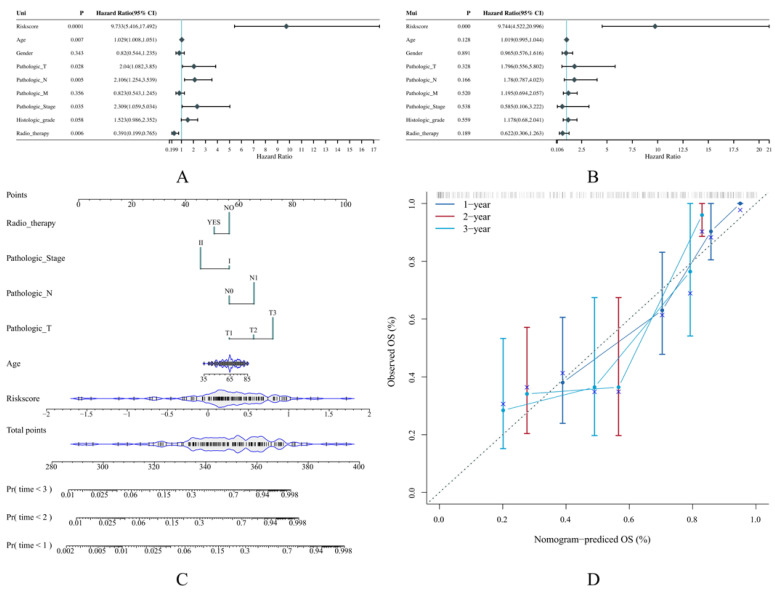
The establishment of a predictive nomogram according to our risk model. (**A**,**B**) The forest plot demonstrates the result of univariate and multivariate Cox regression for the risk score and other clinical factors such as age and gender. (**C**,**D**) The nomogram and its calibration curve that contains prognosis-related factors identified from univariate Cox regression. The dashed line in the calibration curve is the ideal line representing 100% accuracy. The more closely the ideal line to the curve, the more precise prediction the corresponding model can make.

Prognosis is usually linked to diverse clinical factors such as age, gender, pathological stage, and histological grades. Therefore, we conducted univariate Cox regression to examine if the risk score was prognosis-related, and multivariate Cox regression to see if it was an independent prognostic indicator, both along with clinical factors. Subsequently, it was found that the risk score, age, T stage, N stage, pathological stages, and radiotherapy were prognosis-related, but only the risk score could serve as an independent prognosis indicator (Figure 5A,B).

Then, we constructed a nomogram that integrated the risk score and all the prognosis-related clinical factors, through which the prediction could be carried out by measuring the corresponding points given specific risk scores and other clinical factors were known. The C-index of the nomogram was 0.786 (Figure 5C). As usual, a C-index over 0.7 is thought to be good at classifying various objects, our constructed nomogram should be deemed excellent. The corresponding calibration curves were also plotted to demonstrate the comparison between the observed and the predictive values. It was found that the 1-, 2-, and 3-year predictive results possessed a good agreement with the ideal line, suggesting that our nomogram predicts the OS rate accurately (Figure 5D).

### 3.5. The Risk Score and IRGPI Genes Are Tightly Associated with the Tumor Microenvironment

**Figure 6:**
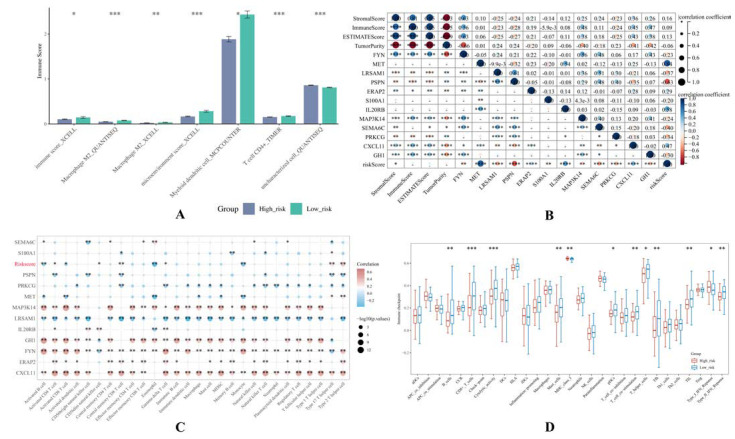
Correlation analysis between the risk score, IRGPI genes, and infiltrating immune cells. (**A**) The histogram demonstrates the 7 immune-related scores which are statistically different in the high- and low-risk groups. (**B**) The heatmap demonstrates the correlation between the risk score, IRGPI genes, immune score, stromal score, ESTIMATE score, and Tumor Purity value. The black circles represent the definite value of Spearman coefficients, and the color bar represents positivity or negativity. The deeper color in blue means the more negative the Spearman coefficient is. The deeper color in red means the more positive the Spearman coefficient is. (**C**) The bubble matrix demonstrates the summary of the correlation between the risk score, IRGPI genes, and 28 infiltrating immune cell types. (**D**) The box plot demonstrates the differences in immune cell infiltration in high- and low-risk groups.

Research in the recent decade has suggested that the tumor microenvironment plays an important role in the immunosuppressive phenomenon of pancreatic cancer through various mechanisms, both the well-understood ones such as the limited immune cell infiltration caused by mechanical constraints and the less-known ones in which the complicated crosstalks between the tumor cells, the desmoplastic stroma, and the immune cells significantly inhibits the normal anti-tumorous function of the T cells [40,41,42]. From this point of view, we elucidated the difference in 119 immune-related scores in high- and low-risk groups by integrating 7 immuno-informatical algorithms (i.e., TIMER, CIBERSORT, CIBERSORT-ABS, QUANTISED, MCPCOUNTER, XCELL, EPIC), resulting in 7 statistically significant phenotypes, including immune score, microenvironment score, and macrophage M2, myeloid dendritic cell, CD4+ T cell, and uncharacterized cell (Figure 6A).

Furthermore, we analyzed the correlation between the risk score, IRGPI genes, the stromal cells, and 28 infiltrating immune cell types through a quantitative scoring system that describes the tumor microenvironment conditions via the immune score, stromal score, and ESTIMATE score. The immune score and stromal score are scores based on the degree of enrichment that can be used to evaluate the content of immune cells and stromal cells within the tumor tissue, respectively. The ESTIMATE score is the sum of them. Together, these scores demonstrate the general phenotype of the tumor microenvironment mathematically. Consequently, the risk score was found negatively related to the immune score, and except for S100A1, PSPN, and IL20RB, the other IRGPI genes were significantly related to the aforementioned scores (Figure 6B). Except for LRSAM1, MET, and PRKCG, the rest were positively associated with the immune score, stromal score, and ESTIMATE score. The intratumoral heterogeneity was also explored using the Tumor Purity value, with which CXCL11, ERAP2, FYN, GH1, MAP3K14, and SEMA6C were found negatively correlated, while the rest were positively correlated.

In addition, we investigated the relationship between the risk score, IRGPI genes, and diverse infiltrating immune cell types. It was found that the risk score was negatively associated with activated B cells, activated CD8+ T cells, eosinophils, and monocytes, but positively associated with activated CD4+ T cells, CD56dim natural killer cells, central memory CD8+ T cells, Th17 cells, and Th2 cells (Figure 6C).

The difference in diverse infiltrating immune cell types between the high- and low-risk groups was also investigated. To be more precise, in the high-risk group, the tumor-infiltrating lymphocyte (TIL) is significantly less abundant than that in the low-risk group. Correspondingly, B cells and CD8+ T cells as well as their related activities and partners such as cytolytic activity and T helper cells. are less abundant in the high-risk group than that in the low-risk group, except for MHCI which was almost at the same level between the two groups, and type I IFN receptor which is lower in the high-risk group. The aforementioned findings indicated that the IRGPI-based risk model might serve as a baseline to distinguish different immune cell infiltrating circumstances of individual PAAD patients.

As a supplementary analysis, we also investigated the correlation between the risk score, IRGPI genes, and the well-recognized driver mutation genes including KRAS, TP53, CDKN2A, SMAD4, PIK3CA, MET, STK11, SMARCB1, and JAK3 [4,10,11]. The results suggested that except for TP53, CDKN2A, and SMARCB1, the expression of all the driver mutation genes was tightly associated with the risk score, and merely the expression of CDKN2A is not associated with all the genes comprising the risk model (Supplementary Figure S4). All in all, to a certain extent, these findings supported the idea that the IRGPI might have deeper implications on the carcinogenesis of PAAD.

### 3.6. PAAD Could Be More Precisely Divided into 3 Molecular Subtypes According to the Expression of IRGPI Genes

**Figure 7:**
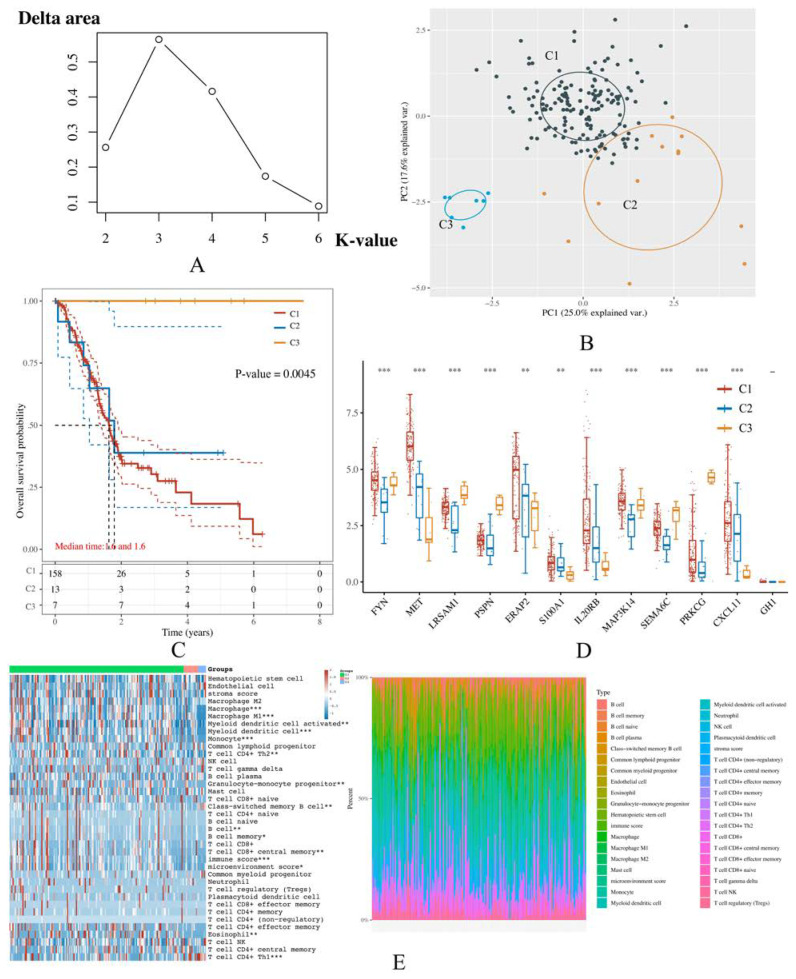
Clustering results for IRGPI-based molecular subtypes. Three molecular subtypes with optimal consensus values were identified and annotated as C1-3. (**A**) The delta area diagrams demonstrate the result of clustering through the K-means algorithm, upon which the most ideal K-value is 3. Therefore, 3 molecular subtypes were deemed most appropriate to be established according to the IRGPI. (**B**) The principal component analysis (PCA), in which the more widely separated each subtype is, the more ideal the consensus clustering result is achieved. As shown in this panel, 3 subtypes were well separated, once again indicating that clustering the samples in this way is suitable. (**C**) The Kaplan Meier (KM) curve of the 3 molecular subtypes is annotated as C1-3. (**D**) The comparison of the IRGPI genes’ expression level between each molecular subtype. (**E**) The heatmap demonstrates different immune-related scores of each molecular subtype on the left and exhibits the abundance of each immune cell type on the right. The *x*-axis represents the TCGA samples involved in the analysis.

As aforementioned, the high- and low-risk groups possessed distinct OS probability and immunotherapy efficacy, it raised our interest in systemically dividing it into more precise molecular subtypes through the K-means clustering algorithm in an unsupervised manner. It was found that when K-value = 3 (i.e., the TCGA samples were grouped into three clusters), the corresponding delta area value reaches its maximum (Figure 7A). At this point, within the principal component analysis (PCA) diagram, it was observed that the samples were well separated (Figure 7B). Therefore, ultimately, 3 molecular subtypes were identified and annotated by C1 (*n* = 157), C2 (*n* = 13), and C3 (*n* = 7). We then investigated the clinical outcomes in these molecular subtypes. Results of the survival analysis suggested that the median survival time of the molecular subtypes is the same, which is 1.6 years (Figure 7C). Furthermore, we examined the expression profiles of the IRGPI genes in each molecular subtype. Consequently, as we expected, except for GH1, they exhibited significant differences in all the subtypes (Figure 7D).

The result of immune-related score estimation suggested that there were significant differences between the molecular subtypes in the immune score, microenvironment score, and many immune cell types, especially Th1 cell, Th2 cell, CD8^+^ central memory T cell, class-switched memory B cell, and memory B cell (Figure 7E).

**Figure 8:**
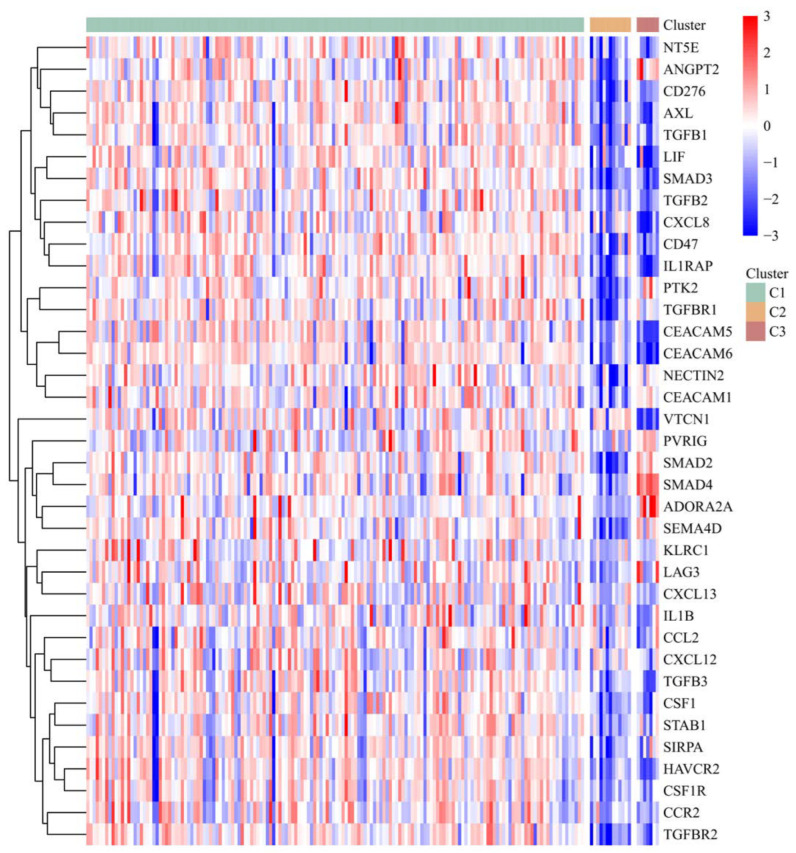
The heatmap displays the expression of the representative genes of the currently FDA-approved immune checkpoints in different IRGPI-based molecular subtypes. The *x*-axis represents the TCGA samples involved in the analysis. The red color indicates a positive correlation, while the blue color indicates the opposite.

For the analysis of the immune checkpoints, we exhaustively screened all the FDA-approved immune checkpoints up to 2021 including SIGLEC15, TIGIT, CD274, HAVCR2, PDCD1, CTLA4, LAG3, PDCD1LG2, CD276, and VTCN1; the result of which showed that in general, subtype 1 demonstrated a more enriched expression profile of these genes, implicating the potential improvement on its immunotherapy. There was also a prominent difference between subtype 2 and subtype 3, implicating that for patients classified into these molecular subtypes, different extents of immunotherapy should be allocated, and different bio-targets should be targeted (Figure 8). Similar to recent in vitro and in vivo studies reported, the immunotherapy targeting B7 family members, especially B7H3 (i.e., CD276) and B7H4 (i.e., VTCN1), exerted promising efficacy on solid tumors [43,44,45,46,47]. Therefore, although the physical barrier in the PAAD tumor microenvironment remains a major obstacle to overcome, our findings may support further optimization of the current immunotherapy strategies.

## 4. Discussion

The long-term survival rates of most cancer types have vastly improved as a result of the evolution of modern medical technology. However, pancreatic adenocarcinoma (PAAD) remains the most fatal malignancy [1,2]. As such, evaluation of clinical outcomes is essential in both financial and humanitarian dimensions. Therefore, we developed a novel immune-related gene prognostic index (IRGPI) consisting of 12 genes including FYN, MET, LRSAM1, PSPN, ERAP2, S100A1, IL20RB, MAP3K14, SEMA6C, PRKCG, CXCL11, and GH1 with a corresponding risk model for OS prediction. In conjunction with other clinical factors, a nomogram was further constructed. Additionally, the risk model was evaluated using external datasets and compared to existing ones, suggesting that our model’s performance was more robust and accurate.

According to the previous study, FYN, LRSAM1, and SEMA6C serve as poor prognostic biomarkers in pancreatic cancer, whereas ERAP2, MET, IL20RB, and CXCL11 indicate improvements. Interestingly, FYN, LRSAM1, IL20RB, CXCL11, S100A1, and MAP3K14 are also well-recognized biomarkers in the prognosis of renal cancer, which is reminiscent of the pancreatic metastasis from renal cell carcinoma in some earlier studies [48,49,50]. Of note, recent advances in the kinome study revealed that multiple emerging kinase targets might play rising stars in the treatment of digestive cancers [51,52,53]. Among them, the FYN proto-oncogene kinase was recently proven to have increased activity in specific cancer types, including pancreatic cancer. FYN-related kinase has been especially highlighted as it not only directly contributes to pancreatic cell proliferation and migration, but also contributes to the development, progression, and maintenance of the inflammatory stroma by promoting desmoplasia, which is in part responsible for resistance to treatment in pancreatic cancer [54,55].

As for GH1, PRKCG, and PSPN, they are not known prognostic biomarkers, but GH1 is still regarded as strongly related to cancer development because of its key role in the stimulation of growth factor secretion (i.e., IGF-1) [56]. PRKCG and PSPN have a function in neurodegenerative diseases [48]. In the present study, additional enrichment analyses revealed that these IRGPI genes are primarily associated with immunological processes and abnormal cell proliferation.

Furthermore, we investigated IRGPI as a risk model in the tumor microenvironment and at the single-gene level. The tumor microenvironment as one of the most critical obstacles in PAAD has been studied extensively. In a literature review, Sofia Liot et. al. summarized the cellular characteristics as exhaustion of anti-cancer cytotoxic T lymphocytes and the infiltration of multiple types of tumor-promoting immune cells [57]. In animal experiments, Clark CE et. al. reported that tumor-associated macrophages, myeloid-derived suppressor cells, and regulatory T cells (Tregs) appear in the early precursor lesions of PC and persist through invasive cancer in the mouse [58]. Interestingly the transcription factors characteristic of Tregs (i.e., FOXP3 and RORγt) were not identified as prognostic factors in the current evaluation, despite their reported persistence in invasive cancer. This might be due to the dual role of these cells in these types of tumors [59], where they have exhibited both pro- and anti-inflammatory effects. In clinical practice, Fukunaga et. al. observed that CD8+ tumor-infiltrating lymphocytes together with CD4+ tumor-infiltrating lymphocytes and dendritic cells were associated with improved postoperative survival [60], whereas the same was not present when CD8+ and CD4+ cells were present alone without the other population. The tumor-associated macrophages found in pancreatic cancer models can also prevent T cells from infiltrating the tumor leading to T cell exclusion in some tumors [40,41,42,61]. Cancer-associated fibroblasts contribute to this effect by molding the extracellular environment to limit T cell infiltration, and by secreting cytokines that can limit effector cell functions [62,63,64,65].

The role of other immune cells should not be discounted either. Dendritic cells have been identified as an independent prognostic factor for PAAD [66], with lower circulating dendritic cell numbers resulting in a worse prognosis [67]. Tumor-associated macrophages also interface with tumor-associated neutrophils, with the overall effect of driving tumor progression through the promotion of hypoxia, vascular remodeling, fibrosis, immunosuppression, and metastasis formation [68]. In short, despite some less-satisfying attempts, randomized clinical trials targeting the tumor microenvironment are still ongoing, and research on crosstalks between the risk score, IRGPI genes, stromal cells, and immune cells is in demand. Therefore, in the present study, starting from evaluating the general appearance of the tumor microenvironment, we qualitatively analyzed the content of stromal cells and 28 infiltrating immune cell types in high- and low-risk groups. We found that these groups were quite distinct from one another, indicating that the IRGPI was potentially connected to the tumor microenvironment. The results of the correlation analysis between the risk score and the tumor microenvironment once again supported such ideas.

## 5. Conclusions 

Based on the IRGPI genes, we clustered PAAD into three distinct molecular subtypes in a more precise way, demonstrating distinct correlations with the expression of current FDA-approved immune checkpoints, and providing a new insight for future precision and translational medicine.

## Data Availability

The datasets presented in this study can be found in online repositories. The names of the repository/repositories and accession number(s) can be found in the article or Supplementary Materials.

## Supplementary Materials

The following supporting information can be downloaded at: https://www.mdpi.com/article/10.3390/cancers14225652/s1, Figure S1: List of the 149 DEIRGs that were significantly related to the OS of PAAD patients. Figure S2: Process of feature gene selection. Figure S3: Expression analysis of IRGPI genes. Figure S4: Correlation between the risk score, IRGPI genes, and driver mutation genes.
