# Supplementary material for: A Novel Immune-Related Gene Prognostic Index (IRGPI) in Pancreatic Adenocarcinoma (PAAD) and Its Implications in the Tumor Microenvironment
Source: Cancers (Basel). 2022 Nov 17;14(22):5652. doi: 10.3390/cancers14225652 (PMC9688924; doi:10.3390/cancers14225652)

	p.value	Hazard ratio
VDR	0.033	1.575(1.037−2.393)
S100A16	0.001	2.003(1.314−3.051)
PPP3CA	0.005	1.824(1.195−2.784)
RAC1	0.002	1.976(1.294−3.018)
S100A10	0.002	1.927(1.264−2.938)
SEMA3C	0.006	1.816(1.190−2.774)
BIRC5	0.014	1.692(1.113−2.573)
NENF	0.013	0.579(0.377−0.891)
BST2	0.019	1.652(1.088−2.509)
BMP4	0.019	1.648(1.087−2.499)
BID	0.046	1.526(1.008−2.308)
CMTM6	0.013	1.699(1.118−2.580)
SEMA7A	0.036	1.564(1.030−2.377)
GBP2	0.037	1.552(1.027−2.347)
OASL	0.015	1.674(1.103−2.541)
IL1RN	0.034	1.568(1.034−2.379)
PLAU	0.011	1.719(1.131−2.612)
RARG	0.011	1.707(1.128−2.584)
PPARG	0.047	1.521(1.005−2.303)
PTK2	0.015	1.678(1.105−2.548)
NRAS	0.020	1.649(1.081−2.516)
PSMD7	0.029	1.589(1.048−2.410)
FYN	0.009	0.571(0.375−0.870)
S100A5	0.033	1.569(1.037−2.376)
NCK1	0.007	1.789(1.172−2.730)
TNFRSF10A	0.009	1.742(1.147−2.644)
TRAF3	0.010	0.578(0.381−0.877)
MET	<0.001	2.135(1.391−3.277)
IL1RAP	0.022	1.631(1.072−2.483)
SP1	0.002	1.914(1.258−2.912)
PPP3CB	0.003	0.531(0.347−0.812)
AREG	0.032	1.575(1.039−2.387)
SDC4	0.038	1.559(1.026−2.369)
S100A14	0.034	1.565(1.034−2.369)
KRAS	0.038	1.551(1.025−2.346)
JAG1	0.005	1.830(1.204−2.782)
LRSAM1	0.004	0.535(0.350−0.818)
GREM1	0.038	1.551(1.025−2.345)
PTHLH	0.016	1.671(1.102−2.533)
IFIH1	0.028	1.591(1.051−2.410)
TGFA	0.035	1.566(1.032−2.377)
CCL22	0.037	0.640(0.421−0.975)
BMP2	0.015	1.682(1.105−2.562)
GDF11	0.009	0.572(0.375−0.872)
DKK1	0.011	1.719(1.131−2.613)
NFYA	0.049	1.521(1.003−2.307)
KITLG	0.045	1.528(1.010−2.311)
S100A2	0.033	1.570(1.037−2.377)
OGFR	0.040	0.645(0.424−0.980)
ANXA6	0.008	0.564(0.369−0.862)
TNFSF10	0.020	1.640(1.080−2.490)
DUOX2	0.039	1.550(1.023−2.347)
SEMA6B	0.042	0.649(0.428−0.985)
RABEP1	0.027	0.623(0.410−0.948)
PDGFC	0.048	1.532(1.003−2.339)
PLCG1	0.016	0.598(0.393−0.910)
CXCL9	0.007	1.779(1.172−2.699)
CXCL10	0.008	1.759(1.161−2.665)
CLDN4	0.027	1.599(1.055−2.424)
S100B	0.016	0.599(0.395−0.910)
PSPN	0.015	0.591(0.388−0.902)
IL18	0.010	1.746(1.146−2.661)
TNFRSF10D	0.025	1.606(1.061−2.430)
ERAP2	<0.001	2.137(1.396−3.272)
PPIA	0.013	1.694(1.116−2.572)
PIK3CB	0.008	1.777(1.160−2.720)
TLR3	0.035	1.565(1.033−2.370)
TNFRSF4	0.025	0.619(0.408−0.941)
MUC4	0.039	1.555(1.022−2.367)
PSME2	0.004	1.836(1.212−2.780)
MUC5AC	0.009	1.735(1.145−2.629)
MX2	0.049	1.518(1.002−2.299)
OSMR	0.033	1.579(1.037−2.403)
FAM3C	0.013	1.696(1.118−2.575)
PLSCR1	0.008	1.764(1.163−2.676)
IL11	0.014	1.691(1.111−2.574)
IL1RL2	0.027	0.625(0.412−0.948)
IL15RA	0.018	1.653(1.090−2.508)
CD1D	0.016	0.597(0.393−0.907)
SEMA4B	0.032	1.572(1.040−2.377)
EBI3	0.014	0.593(0.391−0.900)
FGF19	0.011	1.723(1.132−2.623)
APOM	0.018	0.605(0.398−0.918)
S100A1	0.026	0.622(0.409−0.944)
CETP	0.032	0.633(0.417−0.962)
AQP9	0.037	1.555(1.027−2.354)
IL20RB	0.001	2.038(1.330−3.125)
SEMA3G	0.013	0.588(0.386−0.896)
IL17B	0.045	0.649(0.425−0.990)
ADM	0.021	1.632(1.076−2.474)
FLT4	0.032	0.634(0.418−0.963)
RBP5	0.013	0.589(0.387−0.896)
IL17D	0.009	0.571(0.375−0.869)
TYK2	<0.001	0.462(0.300−0.711)
ACKR4	0.031	1.583(1.042−2.407)
EREG	0.024	1.634(1.067−2.502)
JUND	0.026	0.619(0.406−0.944)
PSMD14	0.036	1.558(1.030−2.356)
EGFR	0.021	1.637(1.077−2.488)
MAVS	<0.001	0.485(0.317−0.741)
IL6R	0.001	0.497(0.326−0.757)
MAP3K14	0.016	0.599(0.395−0.908)
CGB7	0.006	1.799(1.184−2.734)
NTF4	0.049	1.518(1.003−2.297)
CRLF2	0.039	0.648(0.428−0.979)
TOR2A	0.047	0.657(0.434−0.994)
TNFRSF13C	0.042	0.648(0.427−0.984)
SCG2	0.001	0.505(0.332−0.768)
CX3CR1	0.018	0.606(0.400−0.916)
LMBR1L	0.010	0.575(0.377−0.876)
SEMA6C	0.033	0.632(0.414−0.963)
SPP1	0.023	1.621(1.069−2.460)
SSTR1	0.015	0.591(0.387−0.904)
IL13RA2	0.011	0.583(0.384−0.885)
IRF3	0.037	0.642(0.424−0.973)
S100Z	0.024	0.620(0.409−0.939)
JAG2	0.038	0.640(0.420−0.975)
IFNLR1	0.034	0.641(0.424−0.968)
SSTR2	0.011	0.585(0.387−0.885)
TRIM5	0.018	1.653(1.091−2.504)
RFXAP	0.019	0.603(0.395−0.919)
KL	0.004	0.542(0.356−0.824)
UCN3	0.007	0.566(0.374−0.856)
MC1R	0.001	0.499(0.325−0.766)
IL6	0.042	1.536(1.015−2.325)
NPR1	0.011	0.581(0.382−0.883)
SSTR5	0.008	0.570(0.375−0.866)
SFTPA2	0.006	1.795(1.183−2.723)
PRKCG	0.043	0.652(0.431−0.986)
LTB4R	0.009	0.570(0.373−0.870)
SLC22A17	0.002	0.522(0.342−0.794)
PCSK2	0.005	0.554(0.365−0.839)
PCSK1	0.014	0.595(0.393−0.899)
CHGB	0.004	0.542(0.358−0.821)
CMA1	0.014	0.597(0.395−0.903)
PPY	0.035	1.567(1.033−2.378)
CXCL17	0.002	1.939(1.271−2.958)
CXCL11	0.030	1.578(1.045−2.382)
LCN12	0.035	0.638(0.420−0.970)
APOH	0.043	0.654(0.433−0.987)
VTN	0.041	0.650(0.429−0.983)
VGF	0.007	0.556(0.364−0.849)
CHGA	0.001	0.505(0.332−0.766)
PAK6	0.022	0.609(0.399−0.931)
LCN10	0.022	0.608(0.398−0.931)
SHC2	0.029	0.629(0.415−0.954)
GH1	0.036	0.641(0.423−0.971)
SEMA6D	0.020	0.609(0.400−0.926)
ACTA1	0.019	0.602(0.394−0.921)

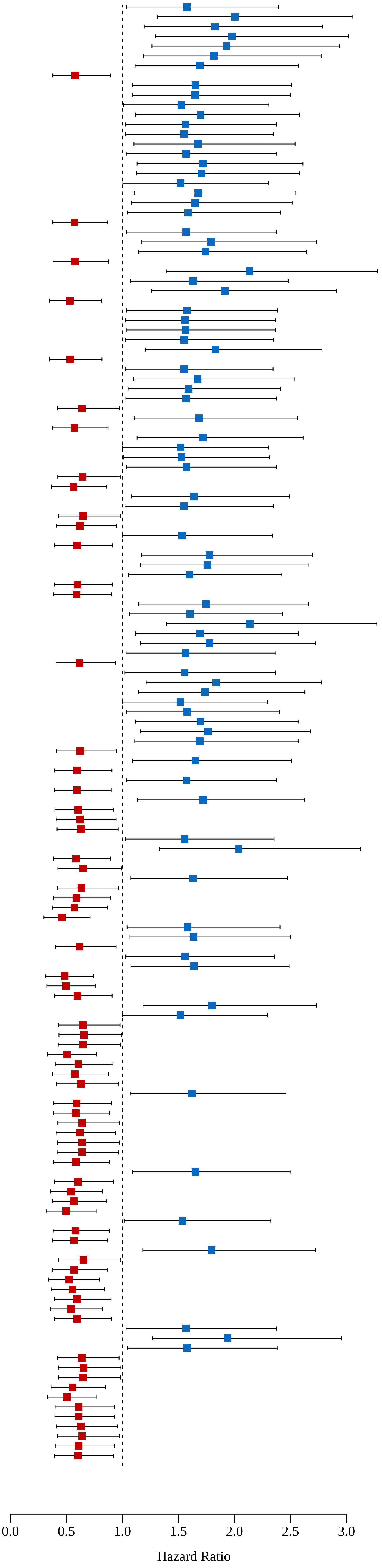

Supplement: Supplementary file 1 [file cancers-14-05652-s001.zip › Supplementary S1.pdf]
