# Supplementary figures and images for: A Novel Immune-Related Gene Prognostic Index (IRGPI) in Pancreatic Adenocarcinoma (PAAD) and Its Implications in the Tumor Microenvironment
Source: Cancers (Basel). 2022 Nov 17;14(22):5652. doi: 10.3390/cancers14225652 (PMC9688924; doi:10.3390/cancers14225652)

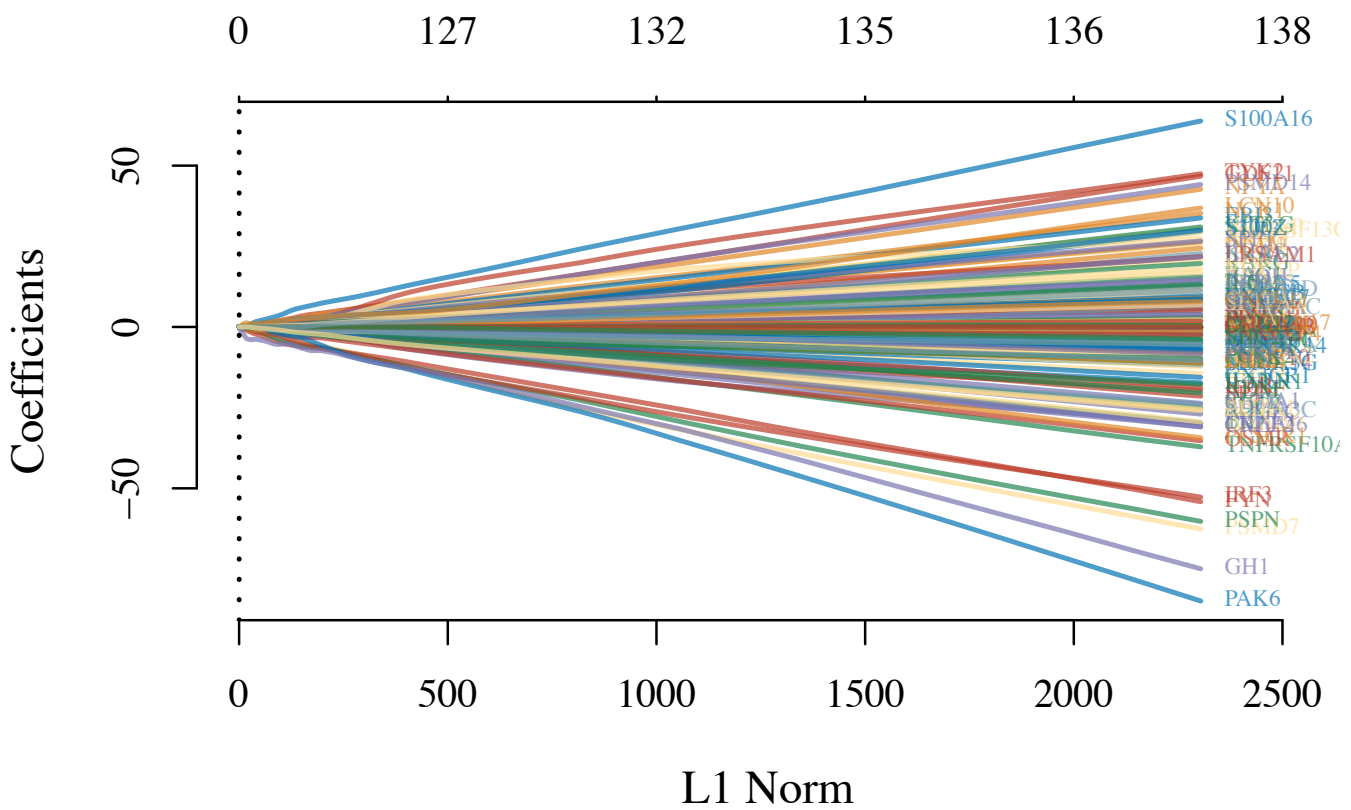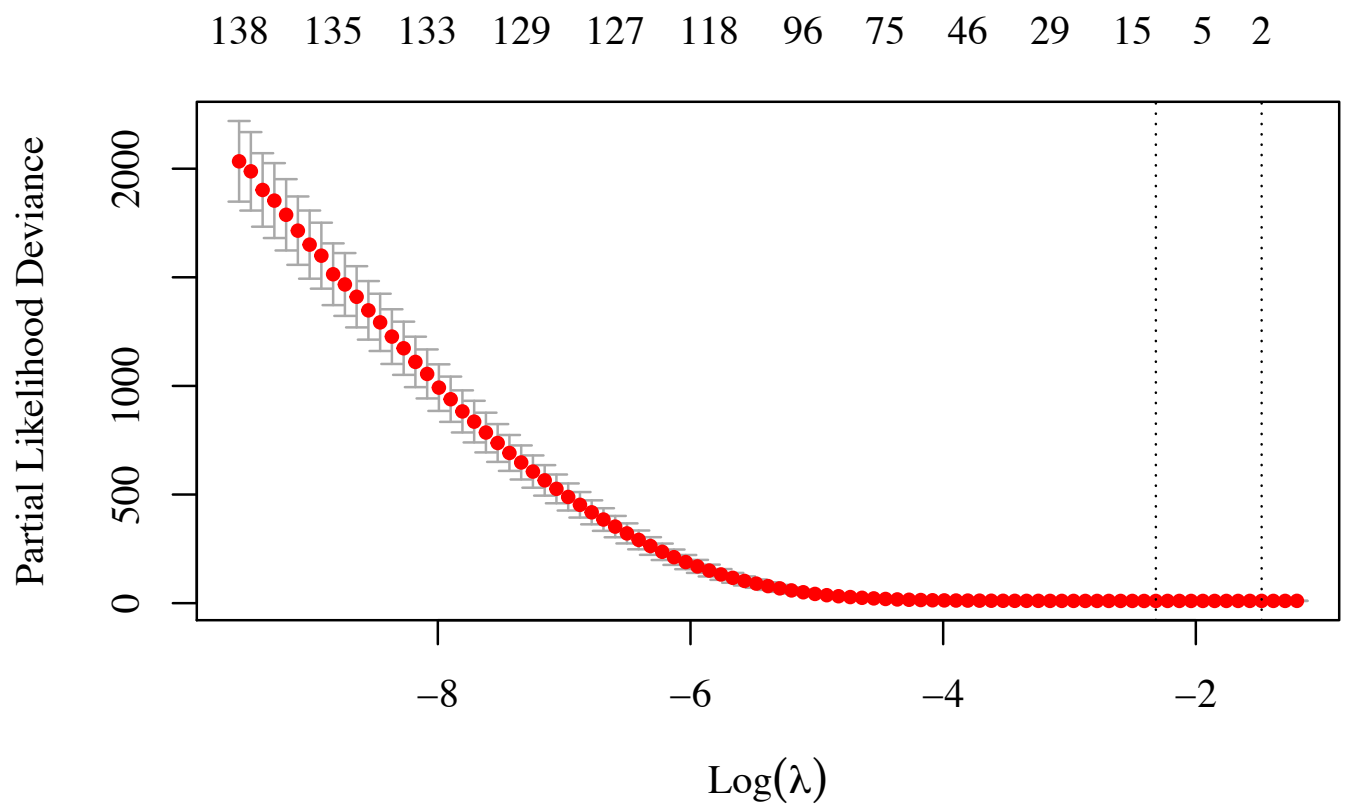

Supplement: Supplementary file 1 [file cancers-14-05652-s001.zip › Supplementary S2.pdf]

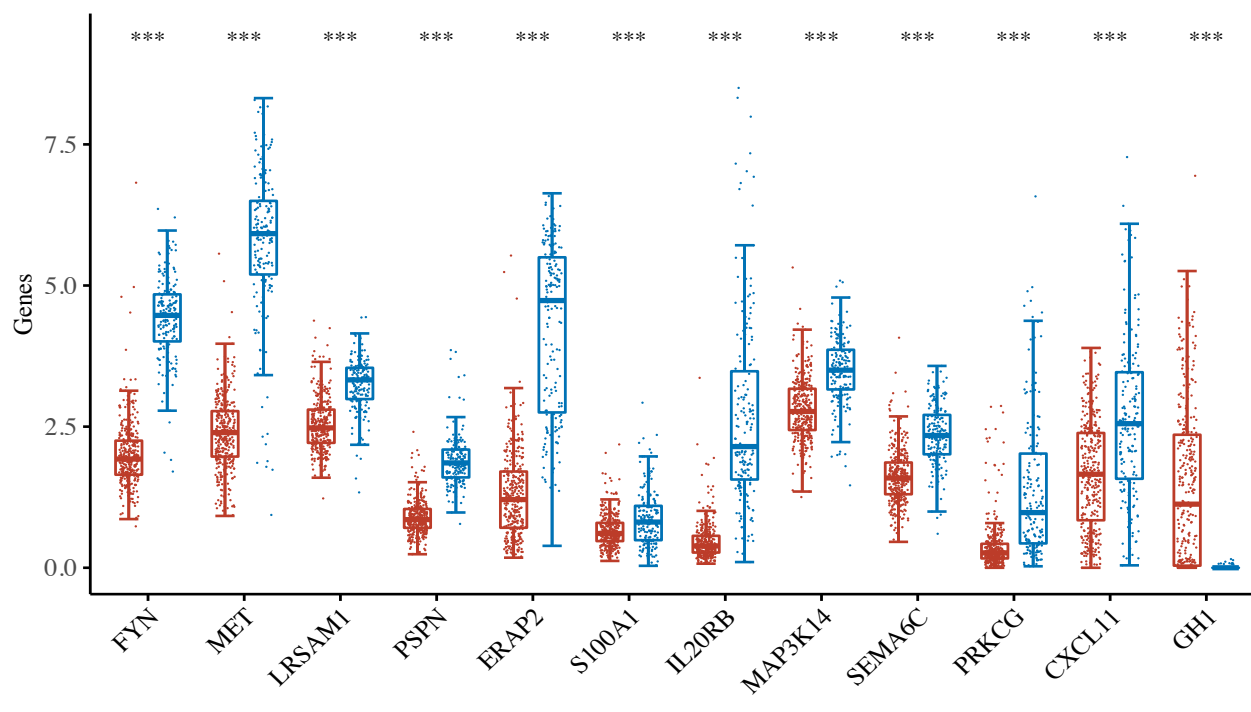

Supplement: Supplementary file 1 [file cancers-14-05652-s001.zip › Supplementary S3.pdf]

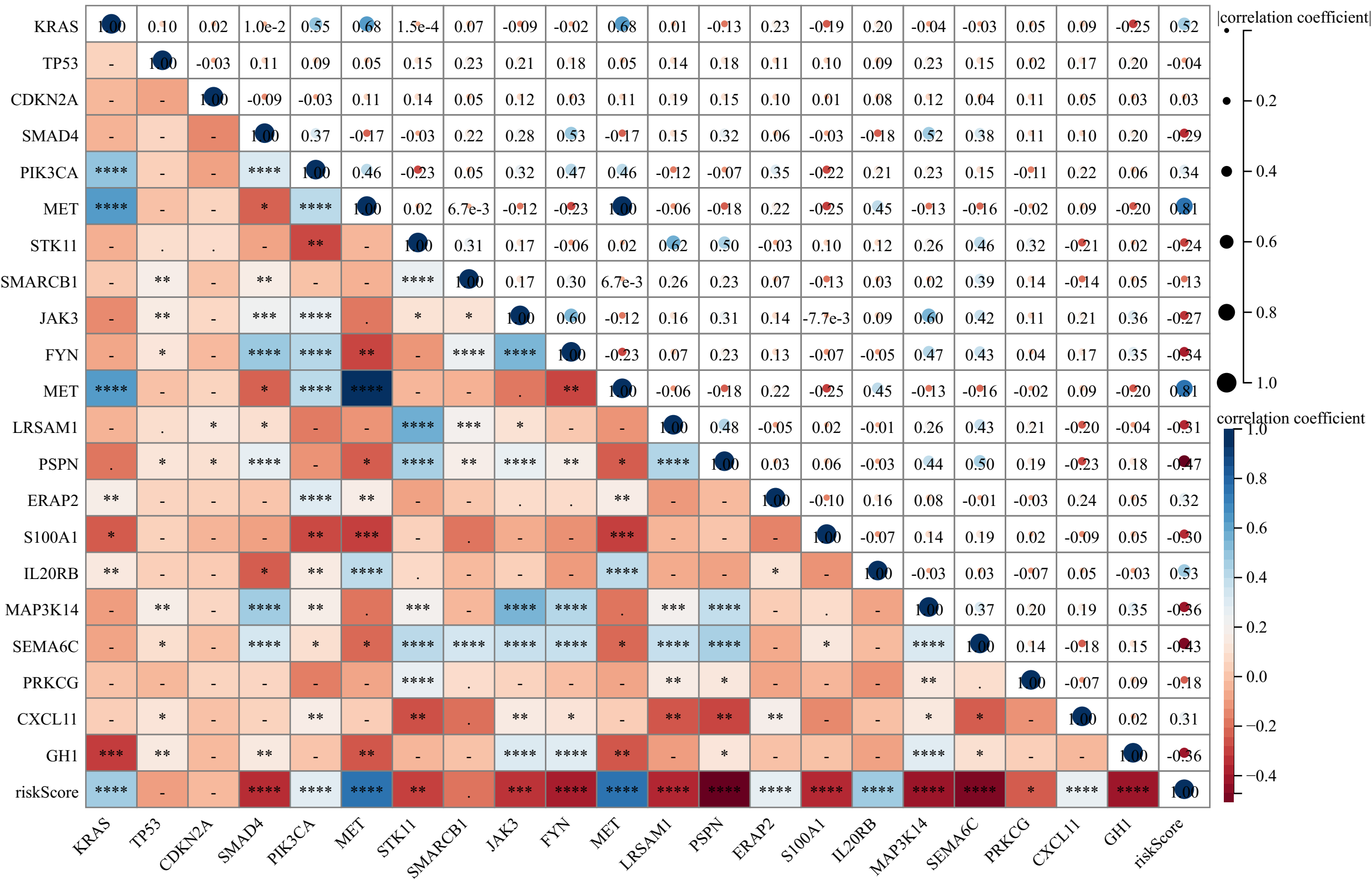

Supplement: Supplementary file 1 [file cancers-14-05652-s001.zip › Supplementary S4.pdf]
